# High abundance of butyrate-producing bacteria in the naso-oropharynx of SARS-CoV-2-infected persons in an African population: implications for low disease severity

**DOI:** 10.1186/s12879-024-09948-z

**Published:** 2024-09-20

**Authors:** Jewelna Akorli, Millicent Opoku, Francis Appiah-Twum, Margaret Sena Akpo, Rahmat Yusif Ismail, Georgina Yaa Kwartemaa Boamah, Elizabeth Obeng-Aboagye, Dina Adu-Asamoah, Irene Owusu Donkor

**Affiliations:** 1 Department of Parasitology, Noguchi Memorial Institute for Medical Research, University of Ghana, Legon-Accra, Ghana; 2 Department of Epidemiology, Noguchi Memorial Institute for Medical Research, University of Ghana, Legon-Accra, Ghana; 3 Department of Virology, Noguchi Memorial Institute for Medical Research, University of Ghana, Legon-Accra, Ghana; 4 Present address Department of Environment and Genetics, School of Agriculture, Biomedicine and Environment, La Trobe University, Bundoora, VIC 3086 Australia

**Keywords:** Naso-oropharyngeal microbiome, SARS-CoV-2, COVID-19, *Bacillota*, *Bacteroidota*, Butyrate producers

## Abstract

**Background:**

The association of the oral microbiome with SARS-CoV-2 infections and disease progression has been documented in European, Asian, and American populations but not in Africa.

**Methods:**

We conducted a study in Ghana to evaluate and compare the naso-oropharyngeal microbiome in SARS-CoV-2-infected and uninfected persons before (pre-vaccine) and after vaccine availability (post-vaccine) in the country. 16S rRNA V3-V4 variable region was sequenced and analysed from DNA extracted from naso-oropharyngeal swabs.

**Results:**

Considering only the infection status, infected and uninfected groups had no difference in their within-group diversity and was evident in the study population pre- and post-vaccine availability. The introduction of vaccines reduced the diversity of the naso-oropharyngeal microbiome particularly among SARS-CoV-2 positive persons and, vaccinated individuals (both infected and uninfected) had higher microbial diversity compared to their unvaccinated counterparts. SARS-CoV-2-positive and -negative individuals were largely compositionally similar varying by 4–7% but considering vaccination*infection statuses, the genetic distance increased to 12% (*P* = 0.003) and was mainly influenced by vaccination. Common among the pre- and post-vaccine samples, *Atopobium* and *Finegoldia* were abundant in infected and uninfected individuals, respectively. Bacteria belonging to major butyrate-producing phyla, *Bacillota* (particularly class *Clostridia*) and *Bacteroidota* showed increased abundance more strikingly in infected individuals before vaccines were available. They reduced significantly after vaccines were introduced into the country with *Fusobacterium* and *Lachnoanaerobaculum* being the only common bacteria between pre-vaccine infected persons and vaccinated individuals, suggesting that natural infection and vaccination correlate with high abundance of short-chain fatty acids.

**Conclusion:**

Our results show, in an African cohort, the abundance of bacteria taxa known for their protective pathophysiological processes, especially during infection, suggesting that this population is protected against severe COVID-19. The immune-related roles of the members of *Bacillota* and *Bacteroidota* that were found associated with infection and vaccination require further studies, and how these may be linked to ethnicity, diet and age. We also recommend expansion of microbiome–disease association studies across Africa to identify possible bacterial-mediated therapeutics for emerging infections.

**Supplementary Information:**

The online version contains supplementary material available at 10.1186/s12879-024-09948-z.

## Background

SARS-CoV-2, which causes COVID-19, emerged at the end of 2019 and spread rapidly through populations. By March 2020, when the WHO declared the disease a pandemic, approximately 118,000 cases and over 4000 deaths had already been recorded across all continents [1]. At the peak of the COVID-19 pandemic, cases and mortality varied widely across the globe. Mortality became the measure by which the burden of COVID-19 was evaluated. Together, Europe and the Americas recorded more than 70% of deaths resulting from COVID-19 [2], and it was initially expected that Africa would be the worst hit by the pandemic. This assumption is because among other factors, health care systems are crucial in determining the general recorded outcomes of infected populations, and Africa is notable for low healthcare delivery [3]. However, the African continent recorded relatively low cases and mortality [2]. Several reasons have been attributed to these statistics, including underreporting, limited testing capacity, high tropical temperatures, increased use of herbal medicine, malaria coinfection with COVID-19 and the frequent use of anti-malarial drugs that inhibit SARS-CoV-2 replication [4, 5].

Host factors also contribute significantly to disease outcome resulting from SARS-CoV-2 infection [6], and identifying these factors is important in explaining individual and population variations in disease epidemiology. The interaction between the host and virus is complex and initiated at the site of entry. For SARS-CoV-2, the naso-oropharyngeal cavity is the major site for viral entry and infection initiation [7]. Here, the binding of the viral receptor to human angiotensin-converting enzyme 2 (*ACE2*) expressed in epithelial cells is crucial for establishing infection [8, 9]. The virus may lodge here for several weeks, replicating in epithelial cells and eliciting a cascade of immune responses that characterize the course of the disease. Low levels of *ACE2* expression in the nasal cavity would therefore lead to decreased viral acquisition [10]. Another cellular activation known to be associated with SARS-CoV-2 infection is the expression of transmembrane serine protease-2 (*TMPRSS2*), which acts as a significant determinant of the entry pathway for the virus; overexpression inhibits viral infection [11]. These findings represent a few examples of our understanding of the intricacy of SARS-CoV-2 infection which could contribute to potential avenues for therapy.

Interactions between bacteria and viruses are common in viral infections. Bacteria may impact viral infectivity and stability, and these interactions could influence how the host responds to viral infections (reviewed in [12]). Oral bacteria could complicate respiratory infections through various suggested mechanisms including promoted adhesion of pathogens to mucosal surfaces and alteration of infection site tissues through periodontal-originating cytokines [13]. The human naso-oropharynx is an environment with a residing community of bacteria, some of which have been reported to be associated with various clinical statuses of COVID-19. Bacterial taxa linked to poor oral hygiene and some opportunistic microbes have been shown to proliferate in COVID-19 patients [14, 15]. Whether this dysbiosis results from the viral infection and facilitates the progress of the disease severity remains unclear. A decrease in gut and oral bacterial diversity has also been linked to elevated levels of proinflammatory cytokines which characterises SARS-CoV-2 [14] and other viral infections, such as hepatitis C virus (HCV) [16] and human immunodeficiency virus (HIV) [17]. Understanding the mechanism of the association of oral bacteria in COVID-19 infection could make useful contributions to our knowledge of COVID-19 pathogenesis and improve care through advanced therapeutics.

In this study, we focused on the naso-oropharyngeal microbiome of SARS-CoV-2-infected and uninfected individuals in an African population with the aim of identifying signature microbes whose known properties may potentially explain the reduced disease severity recorded. We also assessed potential changes in the naso-oropharyngeal microbiome following vaccination, as COVID-19 vaccines have been shown to cause dysbiosis of the gut microbiome [18]. Our study contributes to addressing recent concerns about the neglect of microbiome research in Africa [19, 20], particularly in the face of emerging infections.

## Methods

### Clinical samples

Two sets of clinical samples were used in this study; those obtained during the peak of the COVID-19 pandemic, and the other after vaccination was rolled out in the country. We refer to these samples as ‘pre-vaccine’ and ‘post-vaccine’ sample sets, respectively. For the pre-vaccine set, we randomly selected 49 and 40 previously confirmed SARS-CoV-2 positives and negatives, respectively, from archived naso-oropharyngeal swabs that were tested prior to March 2021 when the AstraZeneca vaccines first became available in Ghana through the UN-partnered COVAX initiative [21, 22]. SARS-CoV-2 detection was performed with standard RT‒qPCR using the Veri-Q PCR 316 COVID-19 Detection Kit (MiCo Biomed Corporation, South Korea), with Ct values < 40 reported as positive. We set a selection criterion to retrieve only naso-oropharyngeal swabs that were collected into and eluted in sterile water. This was to avoid possible loss of microbial richness that could result from the use of viral transport medium (VTM) which may be incorporated with antibiotics.

The post-vaccine samples were swabs collected in June – July 2022 as part of a previous study [23]. These swabs were also placed into 2 mL of sterile water for the same reason already explained and were used for RT‒qPCR viral detection as previously described. Based on the results of the RT-qPCR and the vaccination information collected from participants, samples were grouped into unvaccinated uninfected (UU), unvaccinated infected (UI), vaccinated uninfected (VU) and vaccinated infected (VI).

### Sample processing for microbiome analyses

Total DNA was extracted from 500 µL each of 89 pre-vaccine and 232 post-vaccine samples, respectively, using the ZymoBIOMICS DNA Miniprep kit (Zymo Research, USA). Extractions were performed in batches based on sample availability, especially with the post-vaccine samples which was a running study. To check for potential contamination in downstream analyses, mock (no template) samples were included in each extraction batch. The mock samples totalled 5 and 9 for the pre- and post-vaccine sets, respectively. Eluted DNA was quantified with the Qubit Fluorometer 3.0 (Invitrogen). Based on the DNA quantities obtained and funding availability, 89 pre-vaccine, 68 post-vaccine and all 14 mock samples were prepared for sequencing. Briefly, the bacterial 16S rRNA V3-V4 region was amplified with barcoded primers: 341F: 5’-CCTAYGGGRBGCASCAG- 3’ and 806R: 5’- GGACTACNNGGGTATCTAAT- 3’. Thermal cycling consisted of initial denaturation at 98℃ for 1 min, followed by 30 cycles of denaturation at 98℃ for 10 s, annealing at 50℃ for 30 s, and elongation at 72℃ for 30 s, then a final extension at 72℃ for 5 min. Amplicons with the required size were selected, pooled by equimolar concentrations, end-repaired, A-tailed and ligated with Illumina adapters. Libraries were purified and sequenced.

Eighty (out of 89) pre-vaccine and 65 (out of 68) post-vaccine test samples successfully amplified for 16S rRNA and were processed for sequencing. The pre-vaccine samples consisted of 33 SARS-CoV-2-negative and 47 SARS-CoV-2-positive samples. The post-vaccine samples included 19 unvaccinated uninfected (UU), 7 unvaccinated infected (UI), 19 vaccinated uninfected (VU) and, 20 vaccinated infected (VI). All 14 mock samples were sequenced, whether they amplified or not. The two sets of test samples (including their mocks) were sequenced separately but with the same sequencing criteria on a NovaSeq 6000 platform to generate 250-paired-end reads at a depth of 50 K tags of raw data per sample.

### Sequence processing

Primers and adapters were trimmed off raw reads and resulting sequences < 60 bp long were removed. Reads that contained more than 10% N’s (ambiguous bases) and quality base score ≤ 5 in over 50% of total read length were also filtered out. These resulted in a total of 15,356,104 filtered paired end reads from the pre-vaccine (minimum = 123,189, maximum = 268,461, median = 180,257.0), and 12,707,463 from the post-vaccine set (minimum = 71,449, maximum = 189,728, median = 179,516.0). The sequences obtained from the two sets of samples were merged and processed using customized pipelines and scripts in Quantitative Insights into Microbial Ecology (QIIME2) package version 2023.7 [24] and R-software [25].

The paired sequences were demultiplexed, dereplicated and filtered of chimeras using *dada2* [26] to obtain amplicon sequence variants (ASVs). The ASVs were taxonomically assigned against the SILVA 138.1 database [27] using a customized classifier based on the V3-V4 primers used. Unassigned reads and those identified as Eukaryota, Archaea, Chloroplasts and Mitochondria were removed from the ASV table and representative sequences. A midpoint rooted tree was obtained following the *align-to-tree-MAFFT-fasttree* pipeline under the *q2-phylogeny* plugin in QIIME2. The resulting rooted tree and ASV table were exported into R-software for detection and removal of potential contaminants based on ASV prevalence in the mock samples using *decontam* [28]. A total of 895 ASVs were detected as ‘contaminants’ and excluded from the dataset. Further downstream processing and analyses were performed using R-custom scripts on a *phyloseq* [29] object built with the 145 test samples only. We set an arbitrary filtering criterion to retain taxa that were observed more than once in at least 3% of samples. This identified and removed 4885 ASVs as singletons (taxa represented by one sequence). Because the two sample sets were sequenced separately, Conditional Quantile Regression (ConQuR) was applied on the ASV table to remove batch effect [30]. Rarefaction was performed on this ‘corrected’ data to allow visualization of the adequacy of the sequencing depth to represent the entire microbial contents of all test samples.

### Diversity analyses

Total taxa richness, Shannon‒Wiener, and Simpson ⍺-diversity indices were estimated on rarefied data. The indices were compared among infection and vaccination status using a pairwise Wilcoxon Rank Sum test with a Benjamin-Holchberg (BH) *P*-adjusted correction. Beta (β) diversity analyses were performed in the *microeco* package [31] and were based on Bray‒Curtis dissimilarity [32] and weighted UniFrac distances [33] to allow estimations of variation based on count and taxa phylogenetic distances, respectively. The degree of similarity was estimated using Analysis of Similarity (ANOSIM), while group dispersion was statistically tested with *betadisp*. The amount of variation explained by bacterial composition between test groups was tested with Permutational Multivariate ANOVA (PERMANOVA) accepting as significant adjusted *P*-values < 0.05.

### Differential microbial abundance

Differential taxa abundance analysis was performed at the genus level with *microeco* package [31] in R based on LEfSe [34]. The linear discriminant analysis (LDA) score was set at a threshold of 3 for discriminative features instead of the default of 2 to make the discovery more stringent. Each analysis was bootstrapped 1000 times and *P*-value for test of significance was maintained at the default 0.05.

## Results

### Sequence exploration and statistics

**Fig. 1:**
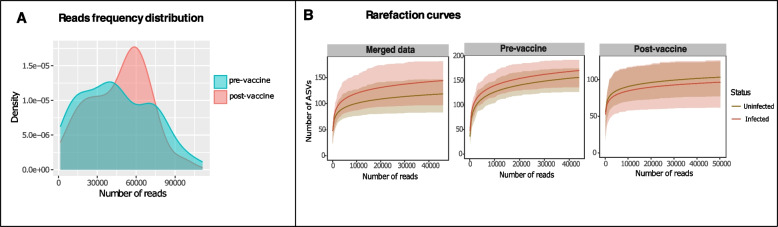
Sequence distribution and rarefaction for sample sets analysed following removal of batch effects. Kernel density plot (**A**) show the reads frequency distribution for pre- and post-vaccine sample sets. Both curves show multimodal distribution and positively skewed distribution. Sub-sampling of resulting sequences produced rarefaction curves (**B**) that depict adequate saturation of the taxa richness from both sample sets

After accounting for and removing batch effects, 6,618,391 reads remained for the merged dataset (Table S1). The average number of reads was 45,644.07; minimum and maximum reads was 1514 and 111,200, respectively. The two datasets had positively skewed distributions but with different read frequencies, indicating that few samples had relatively large number of reads (Fig. 1A). Each dataset was rarefied separately with their respective median read (pre-vaccine = 43,932; post-vaccine = 50,262) providing evidence that the sequencing depth and data processes applied resulted in adequate representations of the total bacterial content per sample and test group without sample size bias (Fig. 1B). The median number of ASVs was higher in the pre-vaccine than in the post-vaccine population (Fig. 1B).

### Within-group microbial diversity increased with vaccination

**Fig. 2:**
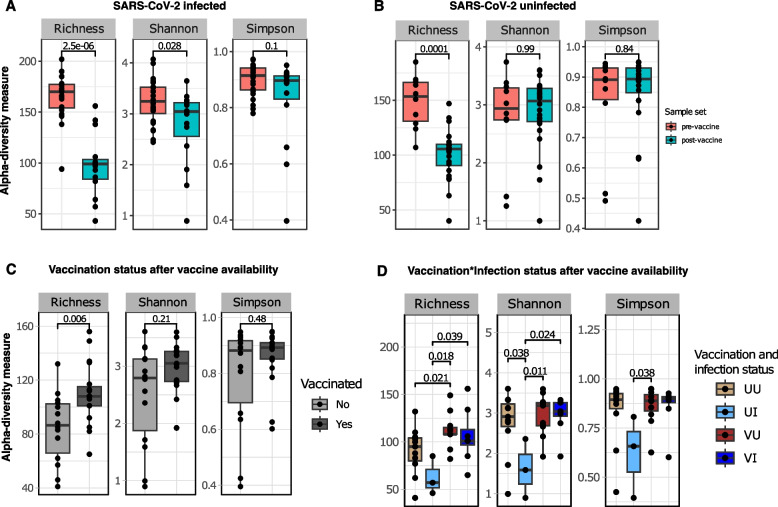
Comparison of taxon richness, Shannon and Simpson alpha (⍺) diversity indices. SARS-CoV-2-infected (**A**) and uninfected (**B**) samples are compared separately between pre-vaccine and post-vaccine sample sets**.** Among the post-vaccine sample set, comparisons are made first between vaccination statuses (without considering infection status) (**C**) and while considering the virus infection status (D). Colored dots represent individual samples, and box plots show first and third quartiles of the distribution. The solid horizontal line in the box shows the median index value per group. Pairwise comparison was achieved with the Wilcoxon test with Benjamin-Holchberg (BH) *P*-adjusted (*P*.adj) correction

Alpha (⍺) diversity did not differ between infection status (positive vs negative) pre- or post-vaccine availability (Fig S1). When infected individuals from the two sample sets were compared, however, species richness and ⍺-diversity estimated by Shannon–Wiener index were higher among the population before the availability of the vaccine (*P.*adj < 0.05) (Fig. 2A). Uninfected individuals in both sample sets only differed in species richness, again being significantly higher pre-vaccine (*P.*adj = 0.0001) (Fig. 2B). Therefore, individual-to-individual microbial diversity was more varied in the study population prior to the introduction of vaccines than after people received COVID-19 vaccination.

We further investigated the post-vaccine dataset to identify whether vaccination influenced within-group diversity. Overall, all vaccinated individuals (infected and infected) showed more variable richness than unvaccinated individuals (*P.*adj = 0.006), but other ⍺-diversity indices were similar (Fig. 2C), implying that the vaccinated group had many rare species. When infection statuses were considered (vaccination * infection), vaccinated individuals showed more varied richness whether they were positive (VI) or negative (VU) for the virus compared to their unvaccinated counterparts (VU vs UU [*P.*adj = 0.02]; VI vs UI [*P.*adj = 0.04) (Fig. 2D). In addition, Shannon–Wiener index was higher in vaccinated infected (VI) compared to unvaccinated infected individuals (UI) (*P*.adj = 0.024), suggesting a significant increase in taxa abundance and richness following vaccination. The impact of vaccination is further confirmed with increased within-group diversity among vaccinated uninfected (VU) compared to unvaccinated infected (UI) individuals (Shannon–Wiener and Simpson indices: *P*.adj < 0.05) (Fig. 2D). The significant Simpson index indicates a change in evenness between microbial diversity associated with being infected without vaccination, and receiving the attenuated virus through vaccines.

### Phylogenetically distinct taxa explain higher diversity between groups

**Fig. 3:**
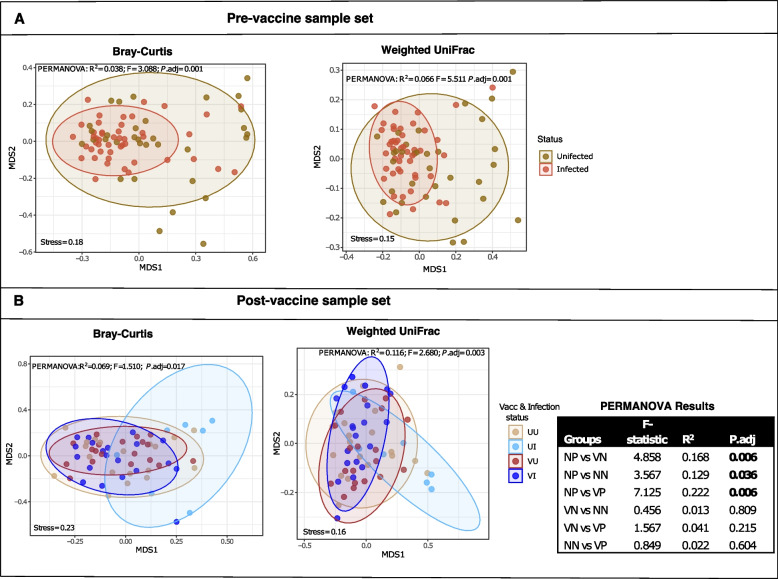
Non-metric dimensional scaling (NMDS) showing sample ordination based on Bray–Curtis dissimilarity and weighted UniFrac phylogenetic distances. The microbial diversity is compared between SARS-CoV-2-positive and -negative individuals before vaccine (**A**) and post-vaccine (**B**) availability considering the vaccination status in the latter sample set. UU = unvaccinated uninfected; VU = vaccinated uninfected; UI = unvaccinated infected and VI = vaccinated infected. Samples are coloured dots and ellipses depict 95% confident intervals of the sample clustering per group. The inserted table are PERMANOVA results for vaccination*infection status groups, showing the *F*-statistic (effect size of variance between compared pair), R^2^ (quantified variation) and *P*. adjusted value (significant results are in bold.)

Principal coordinate analysis (PCoA) was used to visualize the ordination of dissimilarity between sample groups based on Bray‒Curtis (BC), which considers species composition and weighted UniFrac (wUF) distances for bacterial phylogenetic relatedness. Both approaches showed that infected and uninfected groups shared largely similar microbes with few taxa driving little yet significant differences between them. Variation based on bacteria abundance was between 4–7% (PERMANOVA: R^2^ > 0.03; *P.*adj = 0.001) (Fig. 3A). Diversity between infected and uninfected individuals in the post-vaccine population was lower than observed pre-vaccine with no difference between the infection groups (PERMANOVA: R^2^ < 0.015; *P*-adj. > 0.05), unless vaccination statuses were considered (Fig. 3B).

Clustering based on phylogenetic distances always explained a higher variation between test groups than compositional dissimilarity based on counts (Fig. 3). However, there were also significant group dispersions (*betadisp*: Bray–Curtis [*P*.adj = 0.005]; weighted UniFrac [*P*.adj = 0.01]) in the pre-vaccine population suggesting that besides distinct taxa, β-diversity was also influenced by differences in taxa composition within groups in this sample set. Despite this, the microbial differences between SARS-CoV-2-positive and -negative groups were still larger than individual-to-individual differences within each group (⍺-diversity) (ANOSIM: R = 0.17; *P* = 0.001).

### Bacillota and bacteroidota constitute majority of high abundant bacteria

**Fig. 4:**
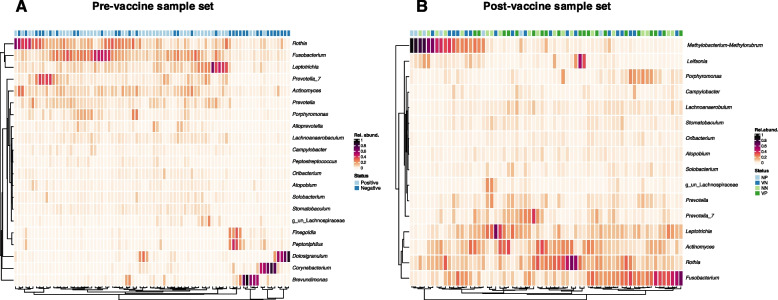
Heatmaps of bacterial genera with average relative abundance > 0.01 in disease groups pre-vaccine availability (**A**) and vaccination*infection status in the post-vaccine (**B**) population**.** Each coloured rectangular block represents the relative abundance of a genus in a sample

The relative abundance of bacterial taxa was observed at the genus level and plotted to show those with > 0.01 total relative abundance. Out of 200 genera identified in the merged dataset, 23 (pre-vaccine = 21; post-vaccine = 16) constituted those with relative abundance > 0.01 and were considered ‘high abundant’ bacteria (Fig. 4). Majority (22 out of 23) of these genera belonged to 5 phyla that together made up > 90% of the bacteria present (Fig S2). Nine genera belonged to *Bacillota* (formerly *Firmicutes*) and four were *Bacteroidota* (formerly *Bacteroidetes*). Twelve of these genera, including *Prevotella*, *Lachnoanaerobaculum*, *Rothia, Actinomyces* and *Fusobacterium,* were common among pre- and post-vaccine populations. *Brevundimonas*, *Corynebacterium*, *Dolosigranulum* and *Finegoldia* were ‘high abundant’ taxa only among pre-vaccination samples, while *Methylobacterium-Methylorubrum* and *Leifsonia* were prevalent among post-vaccine samples (Fig. 4). Distribution of bacteria was generally very patchy, with between 1–5 constituting majority (> 90%) of the microbes in any given sample (Fig. 4).

### Many butyrate-producing bacteria are associated with infection and vaccination

Differentially abundant bacteria that are associated with test groups were also investigated at the genus level since species level classification with short reads of the 16SrRNA variable regions are often inconclusive [35]. The range of identification was not limited to ‘high abundant’ (total average relative abundance > 0.01) but, included low-abundance taxa if they were at least 0.01 relative abundance in one of the tested groups. Although many bacteria could be identified as differentially significant between groups, linear discriminant analyses (LDA) were only plotted for bacteria that satisfied this criterion (Table S2) and had an LDA score ≥ 3 instead of the default LDA score of 2 [34].

**Fig. 5:**
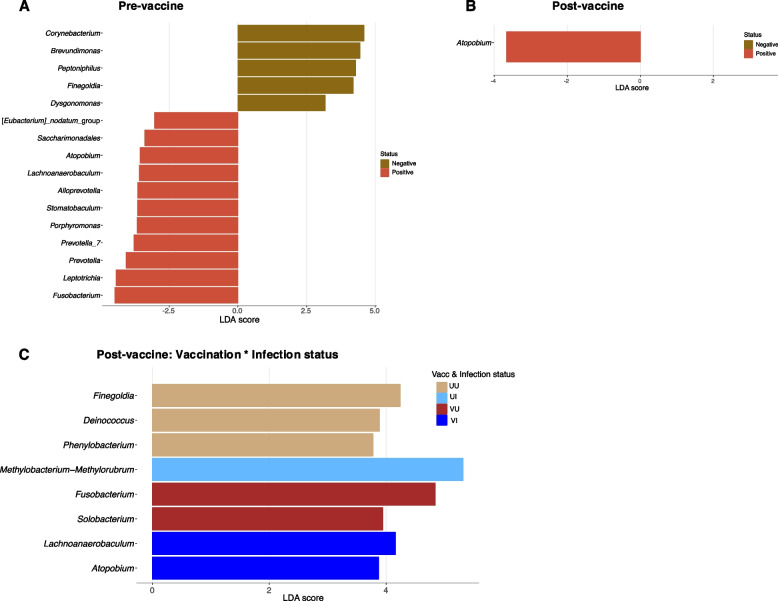
Linear discriminant analysis (LDA) plots for the identification of significant differential microbes between sample groups. The SARS-CoV-2-infected and uninfected persons are compared among pre-vaccine (**A**) and post-vaccine (**B**) populations. Individuals are also compared grouped according to their vaccination*infection status (**C**)

A total of 16 bacterial genera were differentially abundant between SARS-CoV-2-positive and SARS-CoV-2-negative individuals pre-vaccine availability (Fig. 5A). These included 13 of the ‘high abundant’ taxa previously observed (Fig. 4A) and 3 ‘low abundant’ bacteria. Two of these ‘low abundant’ or rare bacteria were differential discriminants of infected individuals (Fig. 5A). About 64% of the differentially abundant bacteria in SARS-CoV-2 positive samples were members of the phylum *Bacteroidota* and *Bacillota* (particularly class *Clostridia*), which are known to be important butyrate producers [36]. Two members of class *Clostridia*; *Finegoldia* and *Peptoniphilus*, were also more abundant in negative individuals.

In the post-vaccine population, however, only *Atopobium* differed between infection status (Fig. 5B), reiterating reduced diversity in this cohort. It was observed to be more abundant in positive individuals as was observed in the pre-vaccine population (Fig. 5A). Accounting for vaccination status, 7 bacteria were found to be differentially prevalent among the test groups (Fig. 5C). Interestingly, *Finegoldia* was again associated with unvaccinated uninfected (UU) individuals confirming its significant correlation with non-infection in the study population with regards to SARS-CoV-2. It is worth noting that *Fusobacterium*, another butyrate producer, was identified as a discriminating microbe, increasing in vaccinated uninfected (VU) persons pre-vaccine and, in positive persons before vaccines were available (Fig. 5A, 5C).

## Discussion

This study is the first to compare the microbial diversity of an African population in relation to infection with SARS-CoV-2 since the pandemic. This study was conducted to address the missing information on how the African microbiome correlated with significant variables that characterised the course of the disease. Two sample sets were obtained in a cross-sectional study design, allowing assessment of the microbial community of the study population at two significant milestones of the COVID-19 pandemic. ie. the peak of the pandemic and after vaccination against SARS-CoV-2 was introduced into the study country. Ghana first received the Oxford-AstraZeneca vaccine, which was primarily rolled-out during the vaccination campaign after which others, including Pfizer BioNTech, Johnson & Johnson and Moderna also became available by June 2022 when the second sample collection was done. While comparing the naso-oropharyngeal microbiome of infected and uninfected within each sample set, we also compared between the two sample sets to conceptualize microbiome changes in the population given the timelines presented. Based on infection status alone, we describe the study population as homogenous with dysbiosis and individual-individual microbial differences evident only when vaccination status is accounted for. While *Atopobium* was significantly associated with SARS-CoV-2 infection, several members of two major butyrate-producing taxa, *Bacillota* and *Bacteroidota*, dominated the naso-oropharyngeal microbiome in both infected and uninfected groups, whether vaccinated or not. Overall, our results are suggestive of a population with protective immunity that may be linked to high abundance of butyrate-producers.

Studies correlating naso-oropharyngeal or oral microbiome with COVI-19 have been conducted in developed countries on other continents including Asia, Europe and North America [14, 15, 37–39]. Their results generally showed significant differential diversity among infected persons and microbial dysbiosis between infected and uninfected groups. While some reported increased diversity [15], others showed a reduction in infected individuals [14, 37]. Contrary to these results, the current study population reports no differences in ⍺-diversity between infected and uninfected persons. These results are indicative of differential microbial responses to infection within and between geographically distant populations.

Human microbiomes differ across continents and, between significantly distant settings within the same geographical location (e.g. country) [40, 41]. It is becoming increasingly evident how the extent of this diversity has been largely underestimated [42, 43]. Among several confounding factors that explain these differences, the ones usually studied in association with microbiomes are ethnicity, age, diet and disease [42, 44–47]. This current study used samples received or collected at a COVID-19 testing centre in Accra, the capital city of Ghana which has the largest population of any region in the country. Information on ethnicity was not collected because it was not relevant for testing suspected cases of COVID-19, and because the samples were not purposely collected for microbiome analyses. However, all study participants were resident in Accra (data collected from questionnaire) and only 4 were foreign nationals. Accra is the most cosmopolitan city in Ghana, and the study participants evident from their names (data not shown) originated from various ethnic groups within the country. Their residency in Accra creates the avenue for several common urbanised lifestyles that can potentially promote homogeneity in their microbiomes.

During the pandemic, it became apparent that COVID-19 severity was linked to the age structure of the population, particularly that of confirmed cases [48–50]. The association of elderly population with COVID-19 severity has also been linked to differences in microbiome structure between old and young [15]. Africa is considered to have the youngest population in the world, having a median age of 19 [51]. More than 70% of Ghana’s population is 35 years and below [52]. The age structure of our study population had a median age of 35 (range 18–68) and 37 years as the median age of confirmed cases (Table S1), which is much younger than reported in oral microbiome studies conducted in Asia and Europe [14, 39]. Only one participant in the post-vaccination group was above 60. Although age data was missing for the pre-vaccination sample set, a previous study conducted in Ghana pre-vaccination demonstrated that 60% of confirmed cases are reported to be in the 20–39 age bracket [53].

Following COVID-19 vaccination, the SARS-CoV-2 spike protein is detectable in plasma and triggers inflammation in epithelial and mucosal sites like the gut [54, 55], and its association with immunity and the gut dysbiosis has been described [55, 56]. Vaccination against COVID-19 has been shown to decrease gut microbial diversity and correlate with stronger immune responses [56]. Similarly, we found our post-vaccination population to have reduced diversity in the naso-oropharynx compared to the study population prior, indicative of positive responses and increased immunity resulting from the vaccines. Given the physical connection and chemical communication between the oral-gut axis [57], we can imply that the dysbiosis observed at both sites are linked, but whether the same microbes are regulated at both sites will have to be confirmed with matched analyses of stool and naso-oropharyngeal swabs.

Comparable to other reports of gut and oral microbiome association with COVID-19, we found several opportunistic periodontitis species correlating with infection. Among these *Prevotella* [14, 38], *Atopobium* [14], *Actinomyces* [58], *Porphyromonas* [15] *Lachnospiraceae*, and *Leptotrichia* [39] significantly increased in infected persons. Although these bacteria were not identified to the species level in the current study, their general lactic acid-producing characteristics and the formation of biofilms [59] support their links with the naso-oropharynx in COVID-19 infections. Particularly, *Atopobium* was found significantly associated with SARS-CoV-2 positivity in both pre- and post-vaccine groups, and even in vaccinated-infected persons. Based on these results, *Atopobium* appears to be a striking microbial marker for detecting SARS-CoV-2 infection in this population, but further studies are required to confirm their predictive efficiency. *Prevotella* is known to be the most abundant genus of the oral microbiome [60] and a notably abundant characteristic microbe in the gut composition of African populations [40, 43] due to high plant and fibre diets [43, 61]. They are also known to be linked to epithelial cytokines and neutrophil recruitment in acute respiratory and chronic diseases [62–64], which would explain their increased abundance in infected persons, primarily in the pre-vaccine population which was presumably novel to the virus. *Prevotella*, *Alloprevotella* and *Leptotrichia*, also have liposaccharide (LPS)-producing properties which are recognised for their involvement in immunoinhibitory pathways [65, 66] but can also be an important host immune stimulant depending on the associated microbial species (reviewed in [67]).

About 61% of all differentially abundant bacteria observed belonged to phyla *Bacillota* (particularly, class *Clostridia*) and *Bacteroidota*, two major butyrate-producing taxa [68–71]. While we find some of these butyrate-producing taxa in uninfected persons, the richness of these microbes was further increased in the presence of the viral infection. Although vaccination reduced species diversity, the bacteria that were differentially abundant in those vaccinated comprised of two *Bacillota*; *Solobacterium* and *Lachnoanaerobaculum*. Butyrate is a short-chain fatty acid that is significant in pathophysiological processes in humans related to inflammatory diseases. They are involved in reducing mucosal inflammation, influencing the fortification of epithelial barriers and promoting the relief of oxidative stress [72]. Butyrate-producing microbes form an essential part of the human gut and oral cavity, are acquired in infancy [73] and are maintained in the adult gut through an increase in fibrous diets [74]. In SARS-CoV-2 infection, butyrate has been shown to be effective in regulating the expression of *ACE2*, proinflammatory cytokines and other genes linked to the progression and disease outcomes of COVID-19 [75] and are predicted to be low in severe COVID-19 [76]. A recent study has directly linked butyrate to reduced cell apoptosis and upregulation of immunity against SARS-CoV-2 infection in experimental mice [77]. Higher abundance of these bacteria in the current study population therefore could be linked to low disease severity and high prevalence of asymptomatic infections [78, 79].

## Conclusion

Our study is limited by the small number of participants, particularly in each group. There is also demographic information about the participants we did not have access to such as their disease progression, number of times they have had the infection, and the time lapse between when they were vaccinated and when they were enrolled into the study. These important confounding factors could have helped explain our results better. However, no similar study has collected all of these in their microbiome-related research, reiterating the importance of standardizing methods for microbiome studies. Despite these the data presented here has shown results that can be used as the basis for further investigating the significance of high abundance of butyrate-producers to disease outcomes, particularly in African populations. The use of other high-throughput techniques such shotgun metagenomics can provide comprehensive functional information of the bacteria associated with infection and vaccination.

## Supplementary Information

Supplementary Material 1. Table S1. Metadata of samples used in this study. For each sample, the re-coded sample ID, SARS-CoV-2 status, sex and test Ct values are shown.

Supplementary Material 2. Table S2. Relative abundance results of discriminant bacteria from performing LEfSe. Only bacteria with ≥0.01 mean abundance in at least one of the groups compared, and with linear discriminant analysis (LDA) score ≥3 were plotted (Fig 5).

Supplementary Material 3. Fig S1. Alpha diversity compared between infection groups without considering vaccination status.

Supplementary Material 4. Fig S2. Average relative abundance of ‘high abundant’ bacterial phyla identified in sample sets. These phyla represented more than 0.01 average relative abundance and constituted more than 60% of all phyla identified.

## Data Availability

All data generated or analysed during this study are included in this published article and its supplementary information file. The demultiplexed sequence reads generated in this study are available in the NCBI SRA database under BioProject PRJNA995054.
