# Supplementary figures and images for: High abundance of butyrate-producing bacteria in the naso-oropharynx of SARS-CoV-2-infected persons in an African population: implications for low disease severity
Source: BMC Infect Dis. 2024 Sep 20;24:1020. doi: 10.1186/s12879-024-09948-z (PMC11414296; doi:10.1186/s12879-024-09948-z)

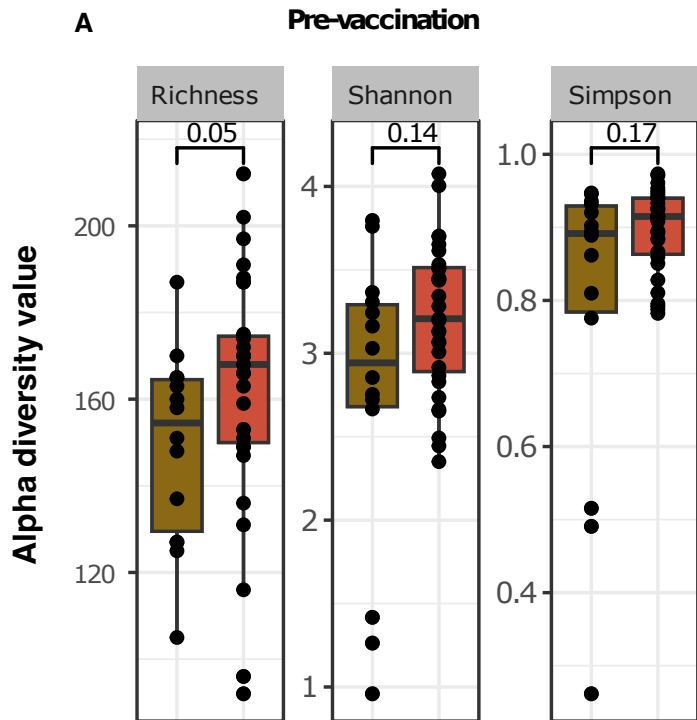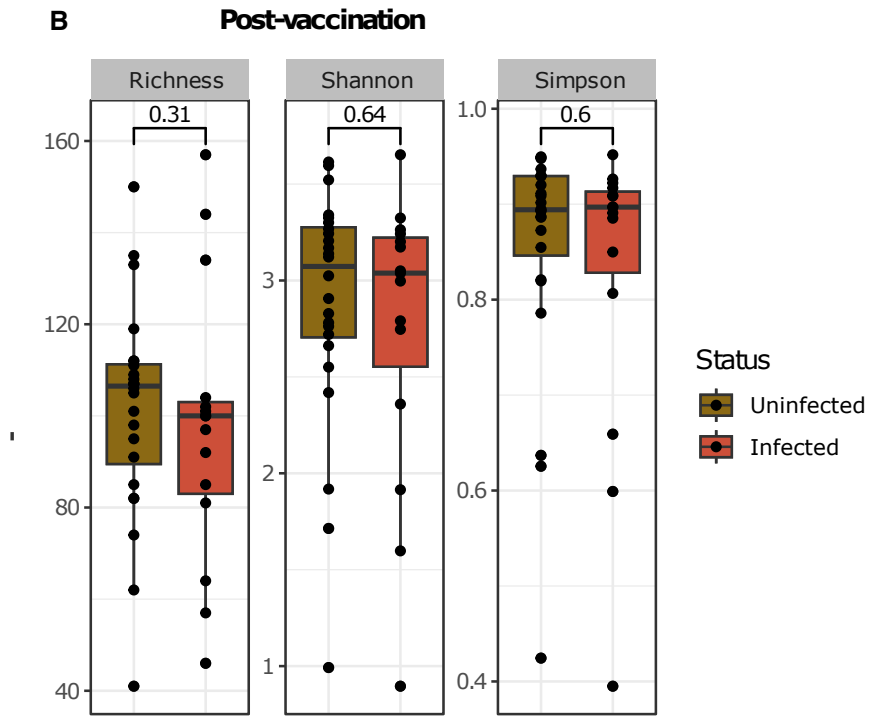

Supplement: Supplementary file 3 — Supplementary Material 3. Fig S1. Alpha diversity compared between infection groups without considering vaccination status. [file 12879_2024_9948_MOESM3_ESM.pdf]

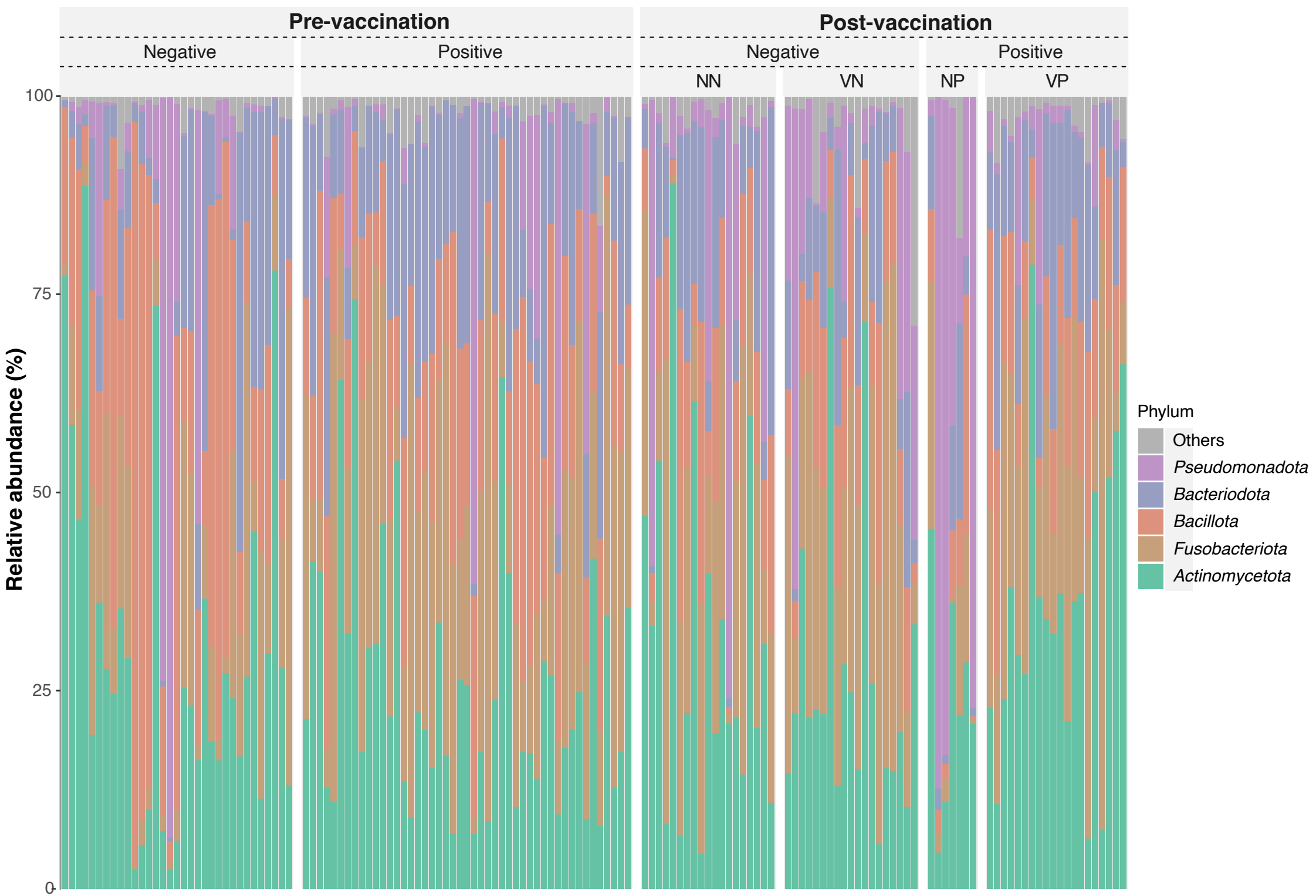

Supplement: Supplementary file 4 — Supplementary Material 4. Fig S2. Average relative abundance of ‘high abundant’ bacterial phyla identified in sample sets. These phyla represented more than 0.01 average relative abundance and constituted more than 60% of all phyla identified. [file 12879_2024_9948_MOESM4_ESM.pdf]
